# Is thyroid status associated with cognitive impairment in elderly patients in China?

**DOI:** 10.1186/s12902-016-0092-z

**Published:** 2016-02-20

**Authors:** Yao Hu, Zhi-cheng Wang, Qi-hao Guo, Wei Cheng, Yan-wen Chen

**Affiliations:** Department of Laboratory Medicine, Huashan Hospital, Shanghai Medical College, Fudan University, Shanghai, 200040 China; Department of Neurology and Institute of Neurology, Huashan Hospital, State Key Laboratory of Medical Neurobiology, Shanghai Medical College, Fudan University, Shanghai, 200040 China

**Keywords:** Alzheimer’s disease, Mild cognitive impairment, Thyroid function, Mini-mental state examination, Memory and executive screening

## Abstract

**Background:**

The relationship between alterations in thyroid function and cognitive deficits has been investigated in several previous studies. Hypo-or hyperthyroidism and, to a lesser extent, subclinical thyroid dysfunction can negatively affect cognitive performance. However, limited data are available on the potential association of thyroid function with mild cognitive impairment (MCI) and Alzheimer’s disease (AD) in the elderly Chinese population.

**Methods:**

In the present study focusing on a population of elderly Chinese individuals ≥ 50 years of age, 77 cognitively normal controls, 64 patients with MCI, and 154 patients diagnosed with AD underwent assessment of thyroid status using thyroid stimulating hormone (TSH), free triiodothyronine (fT3) and free thyroxine (fT4) levels as variables. Cognitive function was evaluated with the aid of comprehensive neuropsychological tests, such as the Mini-Mental State Examination (MMSE) and Memory and Executive Screening (MES).

**Results:**

Overall, 88.1 % of the subjects displayed normal thyroid function, 4.7 % were diagnosed with clinical hypothyroidism, 3.1 % with subclinical hypothyroidism, and 4.1 % with subclinical hyperthyroidism. After adjusting for covariates (age, sex, education years and body mass index), no association was evident between mild cognitive impairment or AD and thyroid dysfunction. However, lower serum TSH was correlated with risk of AD (odds ratio [OR]: 2.78, 95 % confidence interval [95% CI]: 1.11-6.99).

**Conclusion:**

Neither hypothyroidism nor subclinical hyperthyroidism was associated with AD and MCI in this population-based elderly Chinese cohort. Our findings need to be confirmed in a longitudinal study.

**Electronic supplementary material:**

The online version of this article (doi:10.1186/s12902-016-0092-z) contains supplementary material, which is available to authorized users.

## Background

Accumulating evidence has demonstrated that alterations in interrelated endocrine axes are associated with risk of cognitive decline [1]. Thyroid dysfunction may be accentuated or accelerated in elderly people who develop dementia [2]. Consequently, screening for thyroid disease is recommended in dementia workup [3], and the serum thyroid stimulating hormone (TSH) level remains a standard screening tool for the routine assessment of patients presenting with cognitive impairment [3]. In mild cognitive impairment (MCI) and Alzheimer’s disease (AD), subclinical thyroid disease and variations of serum TSH within the reference interval have been increasingly reported, and related to pathogenesis and development of the disease [4–6].

Earlier, the group of van Osch reported that lower TSH is associated with AD [7]. Kalmijn et al. [8] additionally showed that deviation in thyroid hormone levels is a possible risk factor for AD. However, according to previous population-based studies, baseline TSH is not associated with changes over time in cognitive test performance [2, 9, 10], although conflicting results have been obtained on its association with risk of AD [8, 11–13]. Insufficient data are available regarding the relationship between clinical or subclinical thyroid disease and risk of MCI development [14, 15]. Elucidation of thyroid hormone interrelationships in MCI should provide opportunities for earlier intervention in dementia.

The main objective of the current study was to investigate thyroid hormone levels in patients with MCI and AD compared to normal controls, and evaluate their potential association with cognitive function with the aid of comprehensive neuropsychological tests, such as Mini-Mental State Examination (MMSE) and Memory and Executive Screening (MES).

## Methods

### Subjects

A total of 295 participants were recruited, including 77 cognitively normal controls, 64 patients with MCI, and 154 patients with AD. Although normal control subjects were not matched with patients in terms of age and gender, the groups were not statistically different.

Individuals selected for cluster sampling in the Jingansi Community, Shanghai, China, from January 2009 to June 2009 were enrolled as the normal control group using the following inclusion criteria: age range from 50 to 90 years, cognitively normal based on the absence of significant impairment in cognitive functions or activities of daily living (ADL), no memory complaints or difficulties (verified by an informant), Clinical Dementia Rating (CDR) = 0 [16], Hamilton Depression Rating Score [17] less than or equal to 12 on the 17-item scale in the preceding two weeks, and adequate visual and auditory acuity to allow cognitive testing. Exclusion criteria included a history of overt thyroid disease, significant uncontrolled cardiac disease or respiratory failure, insulin-dependent diabetes, uncorrected adrenal cortical insufficiency, chronic liver or renal failure, malignant tumors, cerebrovascular accident, head injury, other neurologic and psychiatric disorders/psychotic features with significant associated cognitive dysfunction, current antipsychotic or anxiolytic medication and other treatments that may have altered thyroid hormone levels, delirium, and recent history of alcohol abuse at the time of assessment. This comparative cohort is an extension of the sample investigated by Guo et al. [18].

In total, 218 patients 50 to 90 years of age were recruited from the Memory Clinic, Huashan Hospital of Fudan University, between October 2010 and 2013. Patients underwent laboratory screening and cranial computed tomography/magnetic resonance imaging scan, with no clinically significant abnormalities in vitamin B12, folic acid, rapid plasma reagin or treponema pallidum particle agglutination.

According to the Peterson criteria [19], MCI is defined in cases of (a) memory complaints and difficulties, as verified by an informant, (b) symptoms lasting more than three months, (c) total score of the Mini Mental State Examination-Chinese version (CMMSE) [20] ≥ cut-off score adjusted for education, objective memory impairment documented based on scoring below the age- and education-adjusted cutoff values in tests of episodic memory, including the Auditory Verbal Learning Test [21], preserved basic activities of daily living (ADL)/minimal impairment in complex instrumental functions, (d) unknown etiology, (e) normal sense of hearing and sight, (f) not meeting diagnostic criteria of dementia based on the National Institute of Neurological and Communicative Disorders and Stroke and the Alzheimer’s Disease and Related Disorders Association (NINCDS-ADRDA) [22].

Probable AD was diagnosed according to the criteria of NINCDS-ADRDA, CDR = 1, onset age ≥ 50 years, no obvious medical, neurological or psychiatric disease or psychological dysfunction, including anxiety and depression, within the previous one month, and no visual or auditory deficits.

### Thyroid hormone measurement and evaluation of thyroid dysfunction

Fasting samples were collected from participants using standard precautions. Serum fT3, fT4 and TSH levels were measured via the chemiluminescent immunoassay performed on an automated assay system (ADVIA Centaur XP, Siemens, Germen). The interassay coefficients of variation (CV %) for fT3, fT4 and TSH were 3.0, 4.0 and 6.4 (level one) and 2.5, 6.0 and 7.1 (level three), respectively. All analyses were conducted using standardized laboratory procedures.

Thyroid dysfunction was assessed based on measurements of fT3, fT4 and TSH levels. According to our laboratory-verified reference ranges, normal serum TSH, fT3 and fT4 intervals were 0.55–4.78 mIU/l, 3.50–6.50 pmol/l and 11.50–22.70 pmol/l, respectively. Cut-off levels for TSH were <0.55 mIU/l for hyperthyroidism and >4.78 mIU/l for hypothyroidism. Cut-off levels for fT4 and fT3 were <11.50 and <3.50 pmol/l for hypothyroidism and >22.70 and >6.50 pmol/l for hyperthyroidism, respectively. Based on hormone levels, patients were classified into four categories: subclinical hyperthyroidism (low serum TSH with normal fT3 and fT4 levels), euthyroidism (normal TSH, fT3 and fT4), subclinical hypothyroidism (high TSH with normal fT3 and fT4) and clinical hypothyroidism (high TSH combined with low fT3 and/or fT4 levels).

### Neuropsychological assessment

All participants were given neuropsychological tests by a certificated professional who was unaware of the cause or diagnosis. A comprehensive neuropsychological battery incorporating memory, language, attention, executive function and visuospatial ability was used, including the Boston Naming Test (BNT) [23], Animal Verbal Fluency Test (VFT) [24], Trail making Test (TMT) [25], Stroop Color Word Test (CWT) [26], Clock-drawing test (CDT) [27], clinical dementia rating scale (CDR) [28], activity of daily living (ADL) [29] and Hamilton depression rating scale (HAMD) [30]. All tests have proven to have good reliability and validity in subjects from a Chinese cultural background.

### Assessment of cognitive function

Cognitive impairment was assessed by means of CMMSE and MES [18]. MMSE is the most widely applied screening tool of mental function in the elderly, routinely used as an outcome measure in clinical trials. The test covers a range of cognitive domains, including orientation to time and place, immediate memory and recall, visuospatial ability, and language. For details on MES, see Additional file 1. There are three indicators of cognition. One sentence with ten main points is remembered three times and free delay recalled twice. The summation of five recall scores is MES-5R, which reflects instant and delayed memory and learning ability. The four subtests of MES-EX include category fluency test, sequential movement tasks, conflicting instructions task and Go/No-go task. This reflects executive function. The total potential score is100, with 50 each for MES-5R and MES-EX. The neuropsychological status of each participant was initially evaluated by a trained rater who was blinded to the thyroid status of all subjects.

## Statistical analysis

Means and standard deviation (SD) were calculated for continuous variables, and significance differences evaluated using one-way analysis of variance (ANOVA) in the three groups (MCI, AD and normal control). Descriptive characteristics of categorical variables were summarized as frequencies, and comparisons among the three groups made using Chi-square tests. Based on low to high serum TSH, all participants were divided into five groups, and logistic regression analyses performed using the fifth quintile as the reference. Logistic regression was used to evaluate the association of thyroid dysfunction with MCI and AD (crude analysis and multivariate adjustment for age, sex and body mass index). All calculated P values were unpaired and two-tailed, and differences considered statistically significant at *P* < 0.05. Bonferroni correction was applied to adjust for multiple comparisons. Data were analyzed using the Statistical Package for Social Sciences (SPSS 13.0, SPSS Inc., USA).

## Ethics statement

Our study was approved by the Huashan Hospital Foundation Ethical Committee. Written informed consent was obtained from all participants. All clinical investigations were conducted according to the principles expressed in the Declaration of Helsinki.

## Results

### Study population

Data from 295 eligible participants were analyzed. The demographic characteristics, neuropsychological assessments and thyroid status of normal control, MCI and AD patients at baseline are presented in Table 1. The mean ages of patients in the normal control, MCI and AD groups were 64.1, 64.3 and 63.5 years, respectively. We observed no significant differences in six demographic variables (age, gender, body mass index, years of education, fT4 and TSH) among the three groups. The means of two variables among the three groups, i.e., MMES and MES scores, were significantly different (*P* < 0.017) after Bonferroni correction. In terms of fT3, we observed significant differences only between AD and normal control (*P* < 0.05), whereby the mean fT3 level of the AD group was higher. Differences in MMSE and MES scores among the three groups were statistically significant. Lowest MMSE and MES scores were observed in the AD groups (*P* < 0.05).

**Table 1:** Characteristics of the study population

Characteristics		Disease status		*P* value
	NC	MCI	AD	
	(*n* = 77)	(*n* = 64)	(*n* = 154)	
Age, years (mean ± SD)	64.1 ± 10.4	64.3 ± 9.6	63.5 ± 9.4	0.822
Gender, No. (%)				0.091
Male	41 (53.2 %)	35 (54.7 %)	86 (55.8 %)	
Female	36 (46.8 %)	29 (45.3 %)	68 (44.2 %)	
BMI, kg/m2(mean ± SD)	23.8 ± 3.1	23.4 ± 3.2	23.4 ± 3.1	0.081
MMSE Score (mean ± SD)	28.0 ± 1.4	26.1 ± 1.8	14.9 ± 5.9	<0.017^a^
MES Score (mean ± SD)	84.0 ± 6.2	63.4 ± 10.9	32.8 ± 18.9	<0.017^b^
Education, years (mean ± SD)	10.1 ± 3.5	9.4 ± 3.5	8.9 ± 4.2	0.094
fT3, pmol/L (mean ± SD)	4.4 ± 0.5	4.5 ± 0.6	4.6 ± 0.5	0.032^c^
fT4, pmol/L (mean ± SD)	15.2 ± 2.2	15.0 ± 1.8	14.8 ± 2.1	0.295
TSH, mU/L (mean ± SD)	2.0 ± 1.0	2.0 ± 1.5	1.8 ± 1.4	0.359

*NC* normal control, *BMI* body mass index

^a^compared with normal control after Bonferroni correction, MCI vs NC *P* < 0.001; AD vs NC *P* < 0.001; AD vs MCI *P* < 0.001

^b^compared with normal control after Bonferroni correction, MCI vs NC *P* < 0.001; AD vs NC *P* < 0.001; AD vs MCI *P* < 0.001

^c^MCI vs NC *P* = 0.310; AD vs NC *P* = 0.011; AD vs MCI *P* = 0.220

Pearson’s correlation analysis was performed to evaluate the relationships among serum thyroid hormone levels, age, education years and cognitive function test scores in all groups (data not shown). Serum fT4 levels were inversely associated with education years in the normal control (γ = -0.26, *P* < 0.05). No significant correlations were evident among serum thyroid hormone levels, age, body mass index, education years and cognitive function test scores in MCI patients. Serum fT3 level was inversely associated with age in AD patients (γ = -0.21, *P* < 0.05). Similar associations were found between serum TSH levels and MMSE and MES scores in AD patients (γ = -0.21, *P* < 0.05; γ = -0.21, *P* < 0.05, respectively). Associations of serum TSH levels with cognitive function test scores in AD patients are presented in Table 2.

**Table 2:** Association of serum thyroid stimulating hormone with cognitive function test scores^a^ in AD patients

Variable	Cognitive function test scores					
	MMSE			MES		
	β(SE)	*P* value	95 % CI for B	β(SE)	*P* value	95 % CI for B
TSH	-0.20(0.34)	0.012	-1.52--0.19	-0.20 (1.09)	0.012	-4.97--0.63

^a^Linear regression models adjusted for age, sex, years of education and BMI

Based on serum TSH, all patients were divided into five groups. Compared with the fifth quintile, participants in the lowest and second lowest quintiles of TSH presented odds ratio (OR) for AD of 2.78 (95 % confidence interval [95% CI], 1.11–6.99) and 2.58 (95 % CI, 1.04–6.39), respectively. All analyses were adjusted for age, sex, years of education and body mass index. The same analyses on subjects with MCI revealed no significant associations. A summary of logistic regression analyses is presented in Table 3.

**Table 3:** Summary of logistic regression analyses^a^

Variable^b^	β(SE)	*P* value	Exponential (B)	95 % CI for exponential (B)
TSH(1)	1.02(0.47)	0.029	2.78	1.11–6.99
TSH(2)	0.95(0.46)	0.040	2.58	1.04–6.39
TSH(3)	0.51(0.44)	0.252	1.66	0.70–3.94
TSH(4)	0.43(0.43)	0.323	1.54	0.65–3.65

^a^Linear regression models adjusted for age, sex, years of education and BMI

^b^Fifth quintile as the reference

Among the 295 recruited participants, 260 had normal thyroid function (20.8 % with MCI and 52.7 % with AD). Hypothyroidism was diagnosed in 14 individuals (12.3 % with MCI and 57.1 % with AD), while 9 had subclinical hypothyroidism (22.2 % with MCI and 55.6 % with AD). There were 12 cases of subclinical hyperthyroidism, including 6 MCI (50.0 %) and 4 AD (33.3 %) patients. No diagnosis of clinical hyperthyroidism was ascertained. Demographic characteristics and distribution of different covariates among the four groups (clinical hypothyroidism, subclinical hypothyroidism, normal thyroid function and subclinical hyperthroidism) are described in Table 4.

**Table 4:** Demographic and clinical characteristics of participants with normal thyroid function and thyroid dysfunction

Characteristic	Hypothyroidism	Subclinical Hypothyroidism	Normal Thyroid Function	Subclinical Hyperthroidism	P value
	(*n* = 14)	(*n* = 9)	(*n* = 260)	(*n* = 12)	
Cognition, No. (%)					0.974
NC	4 (28.6 %)	2 (22.2 %)	69 (26.5 %)	2 (16.7 %)	
MCI	2 (12.3 %)	2 (22.2 %)	54 (20.8 %)	6 (50.0 %)	
AD	8 (57.1 %)	5 (55.6 %)	137(52.7 %)	4 (33.3 %)	
Age, years (Mean ± SD)	65.9 ± 10.9	62.9 ± 11.7	63.7 ± 9.7	64.1 ± 6.5	0.855
Education, years (Mean ± SD)	11.6 ± 4.8	7.4 ± 3.6	9.2 ± 3.8	10.2 ± 3.3	0.078
Gender, No. (%)					0.401
Male	7 (50.0 %)	3 (33.3 %)	147 (56.5 %)	5 (41.7 %)	
Female	7 (50.0 %)	6 (66.7 %)	113 (43.5 %)	7 (58.3 %)	
BMI, kg/m^2^(Mean ± SD)	23.0 ± 3.2	23.3 ± 3.1	23.2 ± 3.2	23.3 ± 3.2	0.840
MMSE Score (Mean ± SD)	20.3 ± 6.7	17.6 ± 10.2	20.7 ± 7.6	23.3 ± 5.1	0.400
MES Score (Mean ± SD)	52.96 ± 23.3	41.4 ± 30.0	52.9 ± 27.1	59.1 ± 18.1	0.514

*NC* normal control, *BMI* body mass index

Compared with the normal thyroid function group, we found no significant association between MCI and thyroid dysfunction after adjusting the model for covariates of age, sex, education years and body mass index (OR, 0.72, 95 % CI, 0.12–4.27; OR, 1.06, 95 % CI, 0.14–8.00; OR, 0.25, 95 % CI, 0.05–1.30, respectively) (Table 5). Similarly, no association of thyroid dysfunction with AD was observed in the multivariate adjusted model (OR, 1.33, 95 % CI, 0.37–4.79; OR, 1.27, 95 % CI, 0.24–6.99; OR, 1.08, 95 % CI, 0.19–6.13) (Table 5).

**Table 5:** Association of thyroid dysfunction with mild cognitive impairment and Alzheimer’s disease, compared to normal thyroid function

	Model	Odds ratio (95 % CI)
All hypothyroidism (*n* = 14)		
Alzheimer’s Disease (*n* = 145)	Crude	1.01(0.29–3.46)
	Multivariate-adjusted^a^	1.33(0.39–4.79)
Mild cognitive impairment (*n* = 56)	Crude	0.64(0.11–3.62)
	Multivariate-adjusted^a^	0.72(0.12–4.28)
All subclinical hypothyroidism (*n* = 9)		
Alzheimer’s Disease (*n* = 142)	Crude	1.26(0.24–6.66)
	Multivariate-adjusted^a^	1.29(0.24–6.70)
Mild cognitive impairment (*n* = 56)	Crude	1.28(0.17–9.37)
	Multivariate-adjusted^a^	1.06(0.14–8.00)
All subclinical hyperthyroidism (*n* = 12)		
Alzheimer Disease (*n* = 141)	Crude	1.01(0.18–5.64)
	Multivariate-adjusted^a^	1.08(0.19–6.13)
Mild cognitive impairment (*n* = 60)	Crude	0.26(0.05–1.34)
	Multivariate-adjusted^a^	0.25(0.05–1.30)

^a^Multivariate adjustment for age, sex, education years and body mass index

## Discussion

In the current cross-sectional study on elderly participants, we observed no significant association of cognitive impairment with hypothyroidism and subclinical hyperthyroidism after accounting for possible interactions and confounding factors. Our findings are consistent with those of previous studies reporting a lack of association between thyroid dysfunction and cognitive decline [10, 31–33]. However, it must be noted that the earlier reports did not specifically focus on the association of clinical and subclinical hypothyroidism with MCI.

On the other hand, several studies have reported that both higher and lower TSH levels within the reference interval are associated with poor cognitive performance in the absence of clinical thyroid disease [8, 11, 34, 35]. Our findings are in keeping with those of previous studies [8, 11, 35] demonstrating increased risk of AD in elderly persons with lower TSH levels. Further validation of the role of TSH as a predictor of AD is warranted with prospective studies using larger sample sizes.

Alzheimer’s disease (AD) is one of the most common causes of dementia in the elderly, affecting more than 24 million individuals worldwide [36]. By the end of 2011, the elderly population over 60 years of age in China was 185 million, comprising 13.76 % of the overall population. Aging and Alzheimer’s disease (AD) are serious public health concerns and causes of social problems in China. MCI, classically defined as a transitional state between normal cognition and dementia [37], is a prodromal stage of clinical dementia defined by cognitive decline greater than expected for normal aging but preservation of activities of daily living. MCI occurs in ~15 % of elderly patients [38], presenting a potential target group for early identification and intervention of dementia.

While the biological pathways underlying the association of low TSH with AD risk remain to be established, proposed theories include both thyroxine-mediated mechanisms and direct effects of TSH on amyloid-β. AD pathology has been proposed to trigger a reduction in secretion of thyroid releasing hormone (TRH), which functions as a neurotransmitter. TRH is secreted not only from hypothalamus but also other areas of the brain, and its secretion may be decreased in the brain of subjects at risk of cognitive decline [39]. Our data showing that subjects with lower TSH are at higher risk of AD support this theory.

Both clinical and subclinical thyroid dysfunction affect cardiovascular risk [40–42]. In parallel, vascular risk factors have been associated with increased risk for AD [43, 44].

The toxicity of thyroid hormone excess to brain is exerted through direct effects on the processing of cerebral amyloid-β proteins and/or local synthesis and release of acetylcholine from neurons. Thyroid hormone has been shown to regulate gene expression of amyloid-β protein precursor (APP). Low central nervous system thyroid hormone levels may therefore contribute to progression of AD by directly increasing APP expression, and consequently, amyloid-β peptide and protein levels. In parallel, TRH depletion is associated with enhanced phosphorylation of tau protein [45]. Increased oxidative stress and decreased antioxidant metabolites have been detected in hyperthyroid patients [46], and exposure to thyroid hormone reported to enhance neuronal death [47].

Our study has a number of significant weaknesses and strengths. This was a cross-sectional analysis on individuals enrolled from a hospital, preventing us from making causal inferences. However, the current report is one of the first to address the association of thyroid dysfunction with cognitive impairment in the elderly Chinese population, which was assessed using CMMSE and MES that present reliable approaches for evaluation of cognitive function in MCI.

## Conclusion

Our results clearly demonstrate that hypothyroidism and subclinical hyperthyroidism are not associated with AD and MCI. The current findings need to be further validated in a longitudinal study. This report contributes to the growing body of evidence showing that hypothyroidism is not associated with MCI.

## Additional file

Additional file 1:
**Memory and Executive Screening (MES).** (DOC 29 kb)

## Abbreviations

AD: Alzheimer’s disease
fT3: free triiodothyronine
fT4: free thyroxine
MCI: mild cognitive impairment
TSH: thyroid stimulating hormone
