# Supplementary material for: Is thyroid status associated with cognitive impairment in elderly patients in China?
Source: BMC Endocr Disord. 2016 Feb 20;16:11. doi: 10.1186/s12902-016-0092-z (PMC4761141; doi:10.1186/s12902-016-0092-z)
Supplement: Additional file 1: — Memory and Executive Screening (MES). (DOC 29 kb) [file 12902_2016_92_MOESM1_ESM.doc]

**Additional file 1: Memory and Executive Screening (MES)**

Question1. The rater should read out the following sentence and have the subject repeat (Do not let the subject read the sentence). [Li] [Xiao-Ming] has [two] [gray] [puppies], and lives at [No. 58], [He-xi] [town], [Yong-an] [county].There are a total of 10 key points, they are the words in the square brackets. Subject gets 1 point for each key point he/she answers correctly. The subject does not score a point if his/her answer is only partially correct (e.g., Saying “Shao-Ming” instead of “Xiao-Ming”). The subject is allowed to repeat the key points in reverse orders (e.g., saying “Li Xiao-Ming’s two gray puppies are gray”). Repeat the sentence two more times and write down the subject’s answer.

Note: 1) Requires rater to read the sentence continuously. Do not respond to any of the subject’s questions in between. 2) After the third time recalling, inform the subject “Please remember the sentence, I will ask you to repeat it later.” 3) The subject does not need to learn the third time if he/she answered all the key points correctly in the first or second time.

Question2. “Please generate all the things you can think of that can be used or seen in the kitchen”, count down 30 seconds and write down all the subject’s answers, even if there are more than 10.

Question3. Conflicting Instructions: “When I tap twice, you tap once, and when I tap once, you tap twice.” In order for the subject to understand the rule, please demonstrate: The practitioner taps once, and the subject should tap twice; the practitioner taps twice, and the subject should tap once. Every tap should be about 2 seconds. Finish the series of number below: 1-1-2-1-2-2-2-1-1-2-1-2-2-1-1. Score method: Mark the ones he/she tapped wrong, minus 1 point for each mistake. Range score is 0–10.

Note: 1) The numbers should be tapped continuously and equally. Finish the task in 30 seconds. 2) Make sure the subject understands the rules completely before starting. Once you begin, tap the numbers equally and do not respond to any interference until the task is done.3) Avoid suggesting to the subject whether to tap or not. 4) The subject only scores when his/her finger touches the table. The tap does not count if his/her finger stops half way. 5) To avoid hurting the subject’s finger while tapping, subject can also choose pounding the table instead.

Question4 Short delayed recall: 4^th^ time.

Rater does not repeat the above sentence and asks subject to recall the previous sentence that was learned before.

Question5. Have the subject use his/her hand to imitate the following action one hand at a time. Imitate every action once.

Step 1: Subject uses dominant hand to imitate the action, and complete with single hand. Subject scores 2 points if correctly done.

Step 2: If the action is not imitated correctly, the practitioner can repeat the action one more time. Subject scores 1 point if correctly done.

Step 3: Subject uses non-dominant hand to imitate the action without the practitioner demonstrating with non-dominant hand. Subject scores 2 points if correctly done.

Step 4: If the action is not imitated correctly with subject’s non-dominant hand, the practitioner can repeat the action one more time with non-dominant hand. Subject scores 1 point if correctly done.

Note the similarity and sequence of the shape of gesture in every movement. 2 points for each action that is completely correct first imitation, and one point if a second repetition is needed (no third repetition).

1) Use thumb to touch other four fingertips in order.

2) Put thumb between index finger and middle finger–scissor shape.

3) Put wrist on same side eye (Telescope-like gesture)–same side ear (Listen-like gesture)–mouth (Drink-like gesture).

4) Do a cross (touch forehead, chest, contralateral shoulder and ipsilateral shoulder by order).

5) Luria action. Instructions: Make a fist, slice down with the edge of your palm, then close your fingers and put the back of your hand flat on the table.

Question6. Inhibitory Control Test (Go/No-go test): “When I tap once, you tap once, and when I tap twice, you don’t tap.” In order for the subject to understand the rule, please demonstrate: The practitioner taps once, and the subject should also tap once; the practitioner taps twice, and the subject should not tap. Start the test after the subject understands the rule. If there are errors in the process, do not remind the subject. Every tap should be about 2 seconds. Finish the series of number below: 1-2-1-2-1-1-2-2-1-1-2-1-2-1-2. [Score method] Mark the ones he/she tapped wrong, minus 1 point for each mistake. Range score is 0–10.

Note: The numbers should be tapped continuously and equally. Do not respond to any of the subject’s questions in between. Finish the task in 30 seconds.

Question7 Long delayed recall: (5^th^ time).

Ask the subject to once again recall the sentence that was learned before.
